# *AluYb8* insertion polymorphism in the* MUTYH* gene impairs mitochondrial DNA maintenance and affects the age of onset of IPF

**DOI:** 10.18632/aging.101793

**Published:** 2019-02-04

**Authors:** Wei Zhou, Jiapeng Sun, Wenwen Guo, Yi Zhuang, Lizhi Xu, Yaping Wang

**Affiliations:** ^1^Department of Medical Genetics, Nanjing University School of Medicine, Nanjing, China; ^2^Department of Respirology, Medical School Affiliated Drum Tower Hospital, Nanjing University, Nanjing, China; ^3^Department of Pathology, The Second Affiliated Hospital of Nanjing Medical University, Nanjing, China; ^4^Jiangsu Key Laboratory of Molecular Medicine, Nanjing University School of Medicine, Nanjing, Jiangsu, China

**Keywords:** polymorphism, *MUTYH*, mitochondrial DNA stabilization, idiopathic pulmonary fibrosis, age of onset

## Abstract

Background: Idiopathic pulmonary fibrosis (IPF) is an age-related fatal disease with an unknown etiology. Increased oxidative stress and mitochondrial dysfunction are thought to be involved in its pathogenesis. However, the effect of the *AluYb8MUTYH* polymorphism on IPF is not known.

Results: The mean age of onset for IPF in patients homozygous for the *AluYb8MUTYH* variant (*P/P*) was 66.5 years old, which was significantly earlier than that in patients with the wild-type (*A/A*, 70.45 years old). For the 97 male IPF patients with lung function data, the FVC% of the* P/P* patients was lower than that of the wild-type (*A/A)* or heterozygous (*A/P*) patients. The laboratory analysis indicated that an increased mtDNA content and impaired mitochondrial quality control were associated with the *P/P* genotype. We also confirmed that *AluYb8* insertion into *MUTYH* caused decreased MUTYH1 expression in lung tissues.

Methods: We compared the lung function of IPF patients and observed the mtDNA content, mtDNA integrity and molecular expression of mitochondrial quality control among subjects with different *AluYb8MUTYH* genotypes. Additionally, immunoblotting and a reporter gene system were used to test whether altered mitochondrial MUTYH1 expression was linked to *AluYb8MUTYH*.

Conclusions: The *AluYb8* insertion polymorphism in *MUTYH* impairs mtDNA stability and affects the age of onset of IPF.

## INTRODUCTION

As one of the most common interstitial lung diseases, idiopathic pulmonary fibrosis (IPF) is defined as a chronic progressive fibrotic lesion with unknown etiology and a histological pattern of usual interstitial pneumonia (UIP). Although the clinical state of IPF can last several years, its prognosis is quite poor, with median survival of 3–5 years from diagnosis. Several risk factors have been implicated in IPF pathogenesis, including smoking, environmental exposures, microbial infections and genetic susceptibility. However, epidemiological data show that aging is the most important risk factor for IPF, suggesting that age-associated changes may predispose individuals to IPF development [1].

Accumulating evidence shows that mitochondria play a key role in the pathogenesis of aging and age-related diseases. Mitochondria produce ATP via oxidative phosphorylation and are the energy-producing organelles of cells. Mitochondria are also the major source of reactive oxygen species (ROS), which can damage biological macromolecules in cells, including lipids, DNA, RNA and proteins. Among the types of ROS-mediated DNA damage, an oxidized form of the guanine base [8-oxo-7, 8-dihydroguanine (8-oxoG)] is considered mutagenic [2]. Mitochondrial DNA (mtDNA) is vulnerable to ROS due to its proximity to the respiratory chain and lack of protective histones. Aging is accompanied by accumulation of 8-oxoG and a reduced oxidative damage repair ability, which can be a driving mechanism for the accumulation of dysfunctional mitochondria and multiple organ injury. Increasing evidence has shown that ROS-mediated DNA damage is involved in a variety of age-related diseases, including sporadic tumors and neurodegenerative, cardiovascular and cerebrovascular diseases [3–5].

To maintain genomic integrity, several biochemical repair pathways have developed in cells. Base excision repair (BER) is the main mechanism that deals with oxidized bases and is initiated by recognition of DNA glycosylation [6]. MUTYH (the human MutY homolog) is a DNA glycosylase enzyme of the BER system that is responsible for removing an adenine that is incorrectly paired with 8-oxoG and leaves an an apurinic/apyrimidine site (AP-site) [7, 8]. Two MUTYH forms [MUTYH 1 (CCDS41320.1, p57) and MUTYH 2 (CCDS41322.1, p60)] are present in human cells. The two MUTYH isoforms are encoded by different transcripts that are grouped into three major categories (named the α, β and γ transcripts) with differing 5’ exons. MUTYH 1 is a product of the α transcript and has a mitochondrial targeting signal (MTS) at its N-terminus, which mainly localizes the isoform to the mitochondria, whereas MUTYH 2 is generated from the β or γ transcript, lacks the MTS and localizes to the nucleus.

We previously described an *AluYb8* element (a transposable short repeat sequence) insertion/deletion polymorphism in the fifteenth intron of the *MUTYH* gene called *AluYb8MUTYH* [9]. According to the presence (P) or absence (A) of the insertion, three genotypes were observed in the human population: homozygous absent (*absence/absence*,* A/A*), homozygous present (*presence/presence*, *P/P*) and heterozygous (*absence/presence*, *A/P*). *AluYb8MUTYH* is a common variant with an allele frequency of 43.2%, and approximately 21% of investigated Chinese individuals have been shown to have the *P/P* genotype for this variation [9]. Additionally, a similar frequency distribution for this polymorphism was detected in healthy Germans by screening a cohort with a small sample size, suggesting that it may also be common variant in the Caucasian population [9]. Using human peripheral blood leukocytes, we found that the *AluYb8* insertion was associated with reduced MUTYH1 protein expression and that the protein was selectively localized in the mitochondria. [10]. Compared to individuals with *A/A* or *A/P*, individuals with the *P/P* genotype had an unstable mtDNA state and decreased mitochondrial activity in their cells, which could affect the occurrence and clinical phenotypes of age-related diseases [9, 11, 12].

In the present study, we extended our investigation of alterations in MUTYH protein expression to individuals carrying different genotypes, evaluated functional impairment of mtDNA maintenance in IPF patients, and examined whether this polymorphism was associated with the occurrence of IPF and affected the prognosis of age-related diseases.

## RESULTS

### The polymorphic distribution of *AluYb8MUTYH* in the IPF patients and healthy controls

The three genotypes were identified by agarose gel electrophoresis of PCR products. Supplementary Table 2 shows the frequencies of the three genotypes detected in the current subjects. The allele frequencies for variant “*P*” were 41.3% and 44.9% in 277 IPF patients and 810 healthy controls, respectively. No significant difference was found between the two groups.

### The pulmonary function test results of the IPF patients

We assessed the correlation between the pulmonary function test results and the *AluYb8MUTYH* genotypes in IPF patients. Pulmonary function test data were collected for 115 hospitalized IPF patients, including 97 males and 18 females. We only compared data from male IPF patients due to the smaller sample size of women. The results showed that the FVC% in the* P/P* patients was significantly lower than that in patients with the *A/A* and *A/P* genotypes. No significant difference was found in FEV_1_% and DLCO% among patients with the three genotypes (Table 1).

**Table 1:** Comparison of pulmonary function test results from IPF patients with different AluYb8MUTYH^*^ genotypes

	Total *n=97*	*A/A n=27*	*A/P n=50*	*P/P n=20*	*P*-value
Age, year	65.5±8.4	65.2±9.7	65.6±8.3	65.5±7.4	
FVC, % predicted	72.6±19.1	74.8±12.6	75.7±20.7	62.1±19.2	0.02
FEV_1_, % predicted	76.1±20.0	77.9±17.5	78.7±21.6	67.0±17.3	0.07
DLOC, % predicted	53.3±21.1	51.8±18.2	56.1±21.9	48.5±22.9	0.36

*All of the tested IPF patients were male. FVC, forced vital capacity; FEV1, forced expiratory volume in 1 s; DLOC, diffusion capacity for carbon monoxide.

### The onset and death ages of IPF patients with different *AluYb8MUTYH* genotypes

We recruited 277 sporadic IPF patients to investigate the relationship between *AluYb8MUTYH* and IPF development. The mean age of occurrence for IPF patients with the *P/P* genotype was 66.5 years old, which was significantly lower than that for patients (70.45 years old) with the *A/A* genotype. Among the 210 IPF patients for whom we obtained follow-up data, 95 patients (45%) died, and the mean survival time from the diagnosis of IPF was 24.6 months. A lower age of death was also observed for the *P/P* patients compared with that of the *A/A* patients (Table 2). However, no significant difference in the survival time of the IPF patients was found among the three *AluYb8MUTYH* genotypes.

**Table 2:** Comparison of the ages of onset and death among IPF patients with different *AluYb8MUTYH* genotypes

Genotypes	Age of onset^*^			Age of death^*^		
	Number	Mean age	*P*-value	Number	Mean age	*P-*value
*A/A*	65	70.5±1.0		32	74.6±1.4	
*A/P*	108	69.7±0.8	0.54	45	71.5±1.3	0.12
*P/P*	37	66.5±1.1	0.01	18	69.4±1.6	0.02

*A comparison was made between the *A/P* and *A/A* or between the *P/P* and *A/A* genotypes using one-way ANOVA, followed by post hoc analysis. The age is shown as the mean ± SD. *P*< 0.05 was considered statistically significant.

### Relationship between the *AluYb8MUTYH* genotypes and the mtDNA content in IPF patients

**Figure 1:**
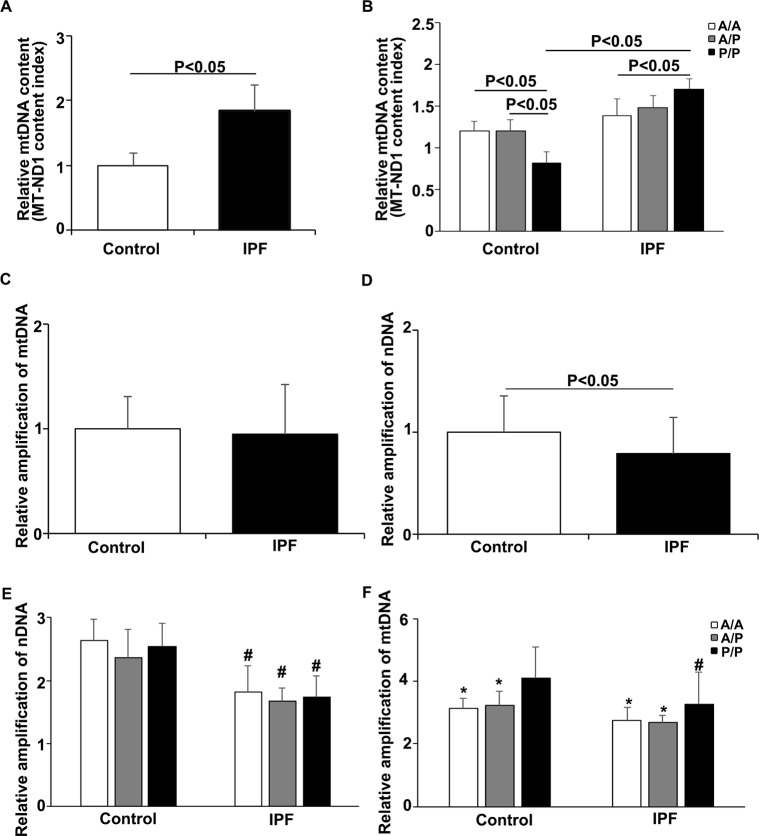
**Relationship between the *AluYb8MUTYH* genotype and mtDNA state in the IPF patients and healthy controls.** (**A**) The *MT-ND1* content index was increased in the IPF patients, *P*<0.05. (**B**) The *MT-ND1* content index of the *P/P* patients was significantly higher than that of the healthy controls with the same genotype, *P*<0.05. (**C**) Relative amplification of mtDNA in the IPF patients and healthy controls. (**D**) Relative amplification of nDNA in the IPF patients and healthy controls. (**E**) Relative amplification of nDNA in patients with different *AluYb8MUTYH* genotypes. (**F**) Relative amplification of mtDNA in patients with different *AluYb8MUTYH* genotypes. * A significant difference compared to *P/P*. # A significant difference compared to the healthy controls with the same genotype, *P*<0.05.

The mtDNA content was examined in peripheral blood cells from 206 patients with IPF and 206 age-matched controls. First, we performed real-time PCR to test the fragments of two genes (*MT-ND1* and *MT-TL1*) as mtDNA markers, and using the *β-actin* as a reference. The results showed that the relative mtDNA content in the IPF patients was significantly higher than that in the healthy controls (Figure 1A and Supplementary Figure 1A). Then, we compared the relative mtDNA content among the IPF patients and the age-matched controls. In the healthy controls, the *P/P* individuals had a decreased mtDNA content compared to that of the *A/A* or heterozygous (*A/P*) individuals (Figure 1B). Unexpectedly, the relative mtDNA content was significantly higher in the IPF patients with *P/P* compared to that of the patients with *A/A*. We also compared the relative mtDNA content between the healthy individuals and IPF patients with the same genotypes and found a significant increase in the mtDNA content only in the *P*/*P* patients, whereas no significant difference was found between the patients and controls with the *A/A* or *A/P* genotype (Figure 1B). The *MT-TL1* results are consistent with those obtained for *MT-ND1* (Supplementary Figure 1B).

The droplet-based digital PCR (ddPCR) method was used for confirmation of the mtDNA copy number analysis with peripheral blood samples randomly selected from the 206 IPF patients and age-matched controls and to verify the correlation of the *AluYb8MUTYH* polymorphism with the mtDNA content. Consistent with the relative quantification of the mtDNA content, we found that the cells from the IPF patients had a significantly higher mtDNA content than those from the age-matched healthy controls (*P* < 0.01) (Supplementary Figure 2A). The controls with the *P/P* genotype had decreased mtDNA contents compared to those of the wild-type (*A/A*) and heterozygote (*A/P*) controls (Supplementary Figure 2B). Additionally, an interesting phenomenon occurred in which the IPF patients with the *P/P* genotype had a significantly increased mtDNA content compared to that of the controls with the same genotype (*P* < 0.05, Supplementary Figure 2B) in this experiment.

### Association of* AluYb8MUTYH* with the accumulation of damaged mtDNA in the IPF patients

**Figure 2:**
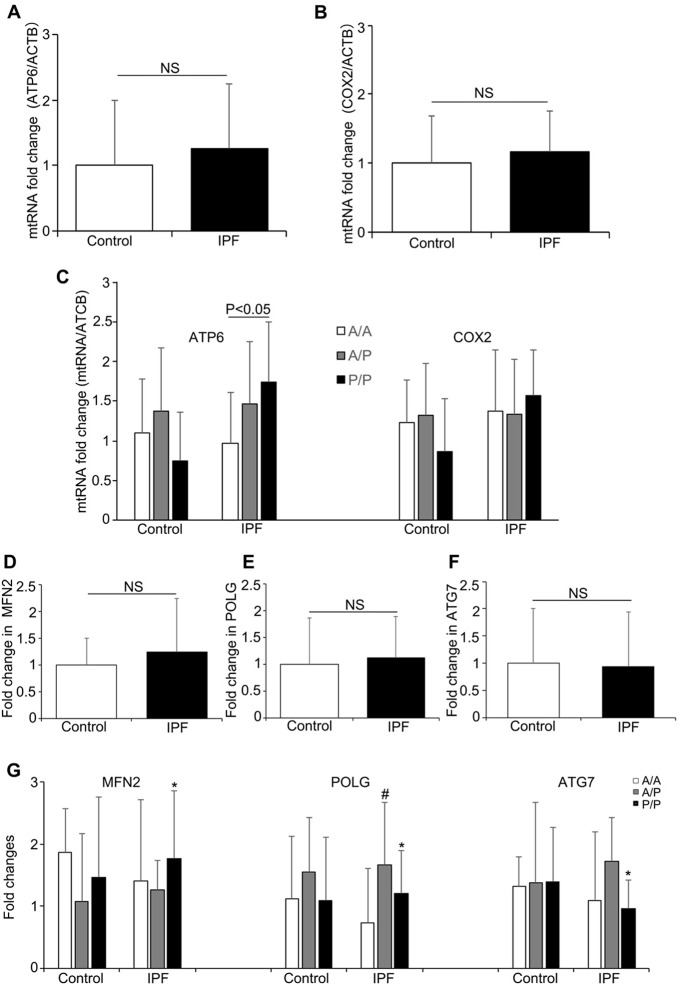
**The mRNA levels of mitochondrial genes and mitochondrial regulation-related genes in the IPF patients and healthy controls.** (**A**) *ATP6* and (**B**) *COX2* mRNA expression in the IPF patients and healthy controls. (**C**) The *ATP6* and *COX2* expression levels in the patients and healthy controls with different A*luYb8MUTYH* genotypes. (**D–F**) *MFN2*, *POLG* and *ATG7* expression in the patients and healthy controls. (**G**) The *MFN2*, *POLG* and *ATG7* mRNA levels among the IPF patients and healthy controls with different *AluYb8MUTYH* genotypes. * A significant difference between IPF patients with the *A/P* and *P/P* genotypes, *P*<0.05. # A significant difference between IPF patients with the *A/A* and *A/P* genotypes, *P*<0.05.

To further investigate the relationship between the *AluYb8* insertion in *MUTYH* and DNA damage in IPF patients, we performed a long-range PCR to analyze the mtDNA and nuclear DNA integrity. As shown in Figure 1C and 1D, the relative amplification of nuclear DNA was significantly lower in the IPF patients when compared with that in the healthy controls. The decreased relative amplification of nuclear DNA was found in IPF patients with the *A/A*, *A/P* and *P/P* genotypes, comparing to the healthy controls with the same genotypes, respectively (Figure 1E), suggesting that an increased oxidative stress exist in the IPF patients. However, no significant difference was found for the relative amplification of mtDNA between IPF patients and healthy controls. Interestingly, the relative amplification of mtDNA was higher in the subjects with *P/P* genotype, either in IPF patients or healthy controls, when compared with the *A/A* or *A/P* genotype (Figure 1F). But, a lower relative amplification of mtDNA was observed in the *P/P* genotype for IPF patients, comparing to the healthy controls with the same genotype. Combining with the result of mtDNA content analysis, we considered that the *AluYb8MUTYH* variant could injure the repair of oxidative damage to mtDNA and cause the accumulation of damaged mtDNA in cells, especially in IPF patients under the severe oxidative stress. To test this hypothesis, we chose *ATP6* and *COX2* in the mtDNA as targets to investigate whether the expression levels of mitochondrial genes exhibited a synchronizing with the variations of mtDNA content and the relative amplification of mtDNA. The results showed no significant differences in mRNA expression of either of the analyzed genes between the IPF patients and healthy controls, but the *ATP6* mRNA expression level was higher in the *P/P* patients than in the *A/A* patients (Figure 2A–2C). Furthermore, we analyzed the transcript levels of the *POLG*, *MFN2* and *ATG7* genes, which regulate mtDNA replication, mitochondrial fusion and mitophagy, respectively. No significant different was found in *POLG*, *MFN2* and *ATG7* mRNA expression between the IPF patients and healthy controls (Figure 2D–2F). In the IPF patients, the *MFN2* expression level in the homozygous *AluYb8MUTYH* group (*P/P*) was significantly higher than that in the heterozygous group (*A/P*), and the *POLG* and *ATG7* mRNA levels in the *P/P* patients were lower than those in the *A/P* patients (Figure 2G). However, no significant different was found in the transcript levels of the three target genes among healthy individuals with the *A/A*, *A/P* and *P/P* genotypes (Figure 2G). These data suggested that the increased level of mtDNA content in the IPF patients with *P/P* genotype could be resulted from an accumulation of impaired mtDNA.

### MUTYH 1 protein expression is highly associated with the *AluYb8MUTYH* genotype

**Figure 3:**
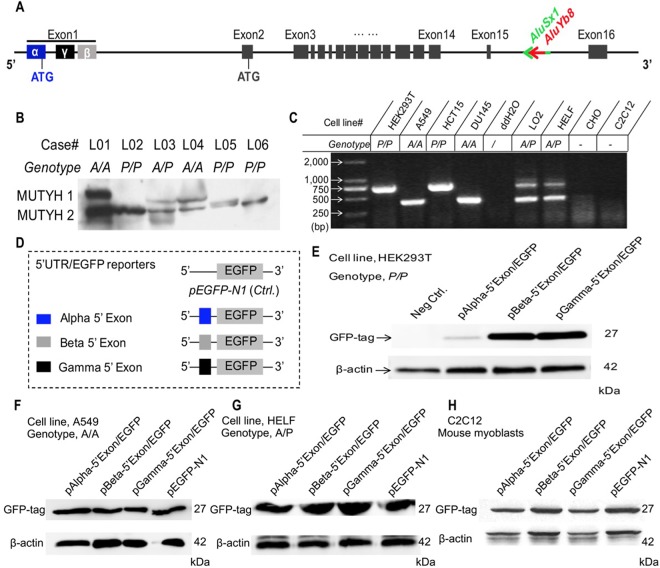
**Association of the* AluYb8* insertion with altered protein expression patterns.** (**A**) Schematic representation of the genomic structure of the *MUTYH* gene. The *AluYb8* element is inserted into existing *AluSx1* sequences in the *MUTYH* 15^th^ intron and is clearly marked by a red arrow. *AluSx1* sequences are depicted with a bright green arrow. Exons are shown with black boxes. (**B**) Representative immunoblotting result showing the altered MUTYH protein expression pattern in human lung tissue cells with the mutant genotype (*P/P*). The two major MUTYH isoforms (MUTYH 1 and MUTYH 2) are indicated. β-actin was used as a protein loading control. The case IDs and their genotypes are shown. (**C**) *AluYb8MUTYH* genotyping of experimental cultured cell lines. (**D**) Schematic representation of the *pEGFP* cloned constructs with different 5’ exon (1^st^ exon) sequences from the *MUTYH* gene. The 5’ exon sequences of the α, β and γ *MUTYH* transcripts are depicted as blue, gray and black boxes, respectively. (**E**–**H**) Representative immunoblotting results for GFP expression in the reporter gene system. The GFP reporter protein of the recombinant protein with the 5’ exon from the α *MUTYH* transcript was observed in the A549 (*A/A* genotype) and HELF (*A/P* genotype) human cell lines and the C2C12 (mouse myoblasts) cell line but not in human cells (HEK293T) with the mutant (*P/P*) genotype. GFP reporters from the recombinant vector with the 5’ exon of the β and γ MUTYH transcripts were expressed in all cultured cells. β-actin was used as a protein loading control.

To uncover whether the MUTYH expression pattern that we previously reported in peripheral blood cells and fibroblasts was also present in lung tissue cells, we obtained snap frozen non-cancerous lung tissues from the tissue bank of the Pathology Department, the Second Affiliated Hospital of Nanjing Medical University, as per the approved guideline. The immunoblotting results showed that MUTYH 1 protein expression was significantly reduced in the mutant (*P/P*) compared to that in either the wild-type (*A/A*) or heterozygous (*A/P*) cells (Figure 3A-B). The decreased MUTYH 1 expression pattern in the lung tissues from patients with the *P/P* genotype was consistent with previous reports in blood cells [10]. Then, we used a reporter gene system to construct recombinant *pEGFP-N1* vectors for verification of the regulatory role of the polymorphism in the 5’-untranslated sequence of the α-type transcript in MUTYH1 expression (Figure 3C-D). We observed that the recombinant *pEGFP-N1* vector with the 5’-untranslated exon sequences of the α-type transcript (*pAlpha-5’Exon/EGFP*) expressed the GFP reporter protein in the wild-type (*A/A*) and heterozygous (*A/P*) human cell lines and rodent cells but exhibited decreased expression in human cells with the *P/P* genotype (Figure 3E). However, the GFP reporters of the recombinant *pEGFP-N1* vector with the 5’ exons of the β- and γ-type MUTYH transcripts (*pBeta-5’Exon/EGFP* and *pGamma-5’Exon/EGFP*, respectively) were highly expressed in all of the cultured cells in this study (Figure 3F–3H). Meanwhile we adopted the reverse transcription quantitative PCR (RT-qPCR) method to determine the mRNA expression levels of the GFP reporter gene in all experimental cells transfected with the recombinant *pEGFP-N1* vectors, with an embedded *neomycin*-encoded gene within the *pEGFP-N1* vector and a housekeeping gene (*β-actin*) used as the references. The mRNA expression analysis results showed no significant differences in transfection efficiency among the three recombinant vectors (comparison of the *neomycin* mRNA levels (Supplementary Figure 4A), under standard transfection conditions, and no significant difference was observed in the GFP mRNA expression levels among these transfected cells (Supplementary Figure 4B). These results indicate that the translation of α-type MUTYH transcripts can be selectively suppressed in cells with the *P/P* genotype and that the *MUTYH* 5’-untranslated exon sequence may participate in regulation of host genes at the translational level.

## DISCUSSION

IPF is a terminal disease that is characterized by extracellular matrix deposition with limited therapeutic choices. The morbidity and mortality of IPF are increasing worldwide and show a positive correlation with aging. Accumulated evidence indicates that oxidative stress and mitochondrial dysfunction are involved in IPF pathogenesis [13]. Furthering understanding of these alterations could provide novel therapeutic targets for IPF and improve quality of life for IPF patients.

The human *MUTYH* gene is located on chromosome 1 and contains 16 exons and primary transcripts (i.e., α, β and γ) [14]. MUTYH 1, which is translated by the α mRNA transcript, initiates the mitochondrial BER pathway by identifying and removing adenines opposite 8-oxoG and 2-hydroxyadenines opposite guanine, thereby preventing the base substitution G:C to T:A [15]. After excision of the mismatched A, an AP site left on the DNA strand generates a single-strand break (SSB), which can be further repaired by endonuclease, DNA polymerase and DNA ligase [16]. Buildup of SSBs in the mtDNA can cause mtDNA depletion, affect DNA polymerase in long-range PCR and activate calpain, thereby executing caspase-independent cell death [17, 18]. This mechanism can be used to explain why the decreased mtDNA content of cells and reduced efficiency of long fragment DNA amplification are associated with an increase in oxidative stress during aging. Conversely, a defect in MUTYH could fail to remove the mismatched adenine and 2-hydroxyadenine during oxidative stress. The AP sites and subsequent SSBs would not be generated, leading to accumulation of oxidative DNA damage in cells. In our study, amplification of long fragment mtDNA was significantly increased in *P/P* patients. This result could be due to loss of mitochondrial MUTYH 1 resulting from the *AluYb8* insertion and a consequent reduction in SSBs. This result also indicated that the increased mtDNA content in *P/P* patients should be associated with accumulation of oxidative damaged mtDNA due to MUTYH 1 dysfunction. In the present study, we also examined MUTYH expression in human lung tissues, and the results verified our previous reports in peripheral blood cells and cultured fibroblasts [10]. In addition, we constructed a recombinant genetic reporter system to test translation regulation of the 5’ exon sequence of the α-type *MUTYH* transcript in cultured cells with the *A/A*, *A/P* and *P/P* genotypes. We confirmed that MUTYH1 expression was greatly inhibited at the translational level in cells with homozygous *AluYb8MUTYH*, which impaired MUTYH1-mediated mtDNA maintenance in *P/P* cells.

IPF is an age-related disease. All of the enrolled patients were ≥ 50 years old, and the oldest was 85 years old. The healthy individuals selected as controls in this work were age-matched. Our results showed that the mtDNA content in the cells from the healthy controls with the* P/P* genotype was lower than that of those with the *A/A* and *A/P* genotypes. This result is in line with our previous reports and reflects the impairment of mtDNA maintenance in the aged healthy controls homozygous for *AluYb8MUTYH* [11]. However, the *P/P* patients showed an increased mtDNA content because transcriptional expression of mtDNA-encoded genes was not upregulated synchronously. We previously showed that *P/P* cells with increased 8-oxoG lesion levels in the genomic DNA had reduced mitochondrial MUTYH 1 protein expression [10]. Sustained low-level oxidative stress can develop with aging and increase the burden of DNA oxidative damage, including 8-oxoG. Cells from individuals with the *P/P* genotype fail to accomplish mtDNA oxidative damage repair because of the mitochondrial MUTYH 1 defect. Thus, mtDNA oxidative damages builds up in the mitochondria and can activate mitochondrial quality control (mtQC), which induces mtDNA degradation and mitophagy. Mitochondria are dynamic organelles, and mtQC is intimately linked to the dynamic behavior of mitochondria and maintenance of cellular homeostasis; thus, mtQC is also an efficient mechanism for preventing the accumulation of impaired mtDNA and maintaining intact mtDNA [19, 20]. The *MFN2*, *POLG* and *ATG7* genes are involved in mtQC and are responsible for regulation of mitochondrial fusion, mtDNA replication and mitophagy, respectively. Compared with the *MFN2*, *POLG* and *ATG7* mRNA levels in the cells of *A/A* patients, the expression levels of the three genes in *A/P* patients could reflect a role for the mtQC mechanism under oxidative stress. The increased *MFN2* expression and decreased *POLG* and *ATG7* expression suggested impairment of mtQC in the *P/P* patients. These results suggested a barrier for mtDNA oxidative damage repair in IPF patients with the *P/P* genotype, which would cause dysregulation of mtQC mechanisms; as a result, enhanced mitochondrial fusion and reduced mitophagy in the cells could lead to an increased mtDNA content and accumulation of mtDNA damage. However, it is still poorly understood the pathogenesis of this disease, further intensive study should be continued to elucidate the mechanism underlying the increase of mtDNA copy number in IPF.

The functions of mitochondria extend well beyond ensuring cellular energy demands. They are also involved in many cellular pathways, including cell proliferation, differentiation, autophagy, and regulation of apoptosis [21]. Dysfunctional mitochondria are even more likely to play a central role in aging and age-related diseases than was previously known. However, the change in the mtDNA level and the mechanisms underlying IPF remain to be elucidated. Ryu C et al. recently reported that the mtDNA concentration was increased in the bronchoalveolar lavage (BAL) and plasma of IPF patients [22]. These patients with high plasma mtDNA concentrations trended towards lower FVC% and reduced event-free survival. However, the origin and characteristics of the extracellular mtDNA in IPF patients have not been described. We observed that *P/P* patients showed lower FVC% than patients with the *A/A* and *A/P* genotypes. Moreover, the *P/P* patients had earlier ages of onset and death for IPF, although different survival times were not observed among IPF patients with the three genotypes.

The main novelty of our work is the relationship between mtDNA maintenance and the *AluYb8MUTYH* genotypes, which has a phenotypic effect on the age-related disease IPF. The *AluYb8MUTYH* polymorphism is a common variant in Chinese and Western populations. IPF patients with the homozygous variant (*P/P*) are associated with an increased mtDNA content and accumulation of mtDNA damage in cells and show earlier onset and death ages for IPF.

## MATERIALS AND METHODS

### Subjects

We recruited 277 patients from Nanjing Drum Tower Hospital Affiliated to Nanjing University School of Medicine with a clinical diagnosis of sporadic IPF from 2002 to 2016. A total of 810 unrelated healthy individuals were enrolled from the same hospital. The details of the IPF diagnosis, enrollment criteria, clinical data acquisition and follow-up information are provided in the Supplementary Methods.

Peripheral venous blood samples were collected from all subjects in EDTA-containing anticoagulant tubes. The demographic characteristics of the study subjects are shown in Supplementary Table 1.

### PCR-based *AluYb8MUTYH* genotyping

Genomic DNA was extracted from the sample cells using the TIANamp Genomic DNA Kit (TIANGEN) according to the manufacturer’s protocol. The *AluYb8* insertion in intron 15 of the *MUTYH* gene was detected by PCR as described in a previous study [9].

### Analysis of the mtDNA content, mtDNA integrity and target gene expression

We used the real-time PCR method to determine the mtDNA content of 206 IPF patients (71 with the *A/A* genotype, 72 with the *A/P* genotype and 63 with the *P/P* genotype) and 206 matched healthy controls (see the Supplementary Methods). A confirmatory experiment was completed using droplet-based digital PCR (ddPCR) (see the Supplementary Methods) to verify the correlation of the *AluYb8MUTYH* polymorphism with the mtDNA content in a proportion of the peripheral blood samples randomly selected from the IPF patients and age-matched controls.

To examine the mtDNA integrity, we performed long-range PCR amplification (see Supplementary Methods) of a specific mtDNA fragment (10 kb) and nDNA fragment (8.7 kb) from 105 IPF patients (35 with the *A/A* genotype, 35 with the *A/P* genotype and 35 with the *P/P* genotype) and 105 matched healthy controls.

We recruited 87 IPF patients (30 with the *A/A* genotype, 30 with the *A/P* genotype and 27 with the *P/P* genotype) and 87 matched healthy controls for analysis of mtDNA coding gene and mitochondrial-related gene expression using the RT-qPCR technique. The mRNA expression of the GFP reporter gene in the transfected cells was detected by the RT-qPCR method. The mRNA levels of the embedded neomycin-encoded gene within the *pEGFP-N1* vector and the housekeeping gene (β-actin) were used as references for the transfection experiments in this study (see the Supplementary Methods).

### Cell culture, plasmid construction and transfection

Human cells (A549, HELF, HEK293T, LO2, DU145 and HCT15) and rodent cells (CHO and C2C12) were grown in suitable complete growth medium (see the Supplementary Methods) and maintained in a humidified incubator at 37°C with 5% (v/v) CO_2_. To investigate whether polymorphic *AluYb8MUTYH* played a regulatory role in the MUTYH 5’-untranslated sequence at the translational level, the three conventional 5’ exon sequences of MUTYH transcripts were cloned, including the 5’-untranslated exon sequences from the α-, β- and γ-type MUTYH transcripts (named α-5’Exon, β-5’Exon and γ-5’Exon, respectively). The three fragments were initially amplified with the designed forward and reverse primers (Supplementary Table 3) and then inserted into the *pEGFP-N1* vector using *Bgl II* and *Pst I* restriction sites. The three *pEGFP-N1*-based recombinant plasmid constructs (named *pAlpha-5’Exon/EGFP*, *pBeta-5’Exon/EGFP*, and *pGamma-5’Exon/EGFP*) were confirmed by sequencing. The cultured cells were grown to 60–80% confluence and transfected with the recombined and control plasmids (*pEGFP-N1*) using an effective transfection reagent (Lipofectamine 3000, Invitrogen). The cells were harvested for analysis after 48 hours in culture.

### Immunoblotting

Tissue or cell lysates were prepared with RIPA lysis buffer (Thermo Scientific). The supernatant was collected and denatured by heating at 95°C for 10 min. Expression of *MUTYH* and the *EGFP* reporter gene was measured by immunoblotting with an anti-MUTYH (BS2535, Bioworld) antibody and GFP-tag (7G9) mouse mAb (Abmart), respectively. The blot was developed with an enhanced chemiluminescent (ECL) detection system (Millipore). Endogenous β-actin expression was used as an internal control.

### Statistical analysis

The statistical calculations were performed using the SPSS Statistics 19.0 software package. The data are expressed as the mean ± SD where indicated. Separate variable comparisons among subjects with different *AluYb8MUTYH* genotypes were conducted with a nonparametric Kruskal-Wallis test or one-way ANOVA, followed by a post hoc analysis. The categorical variables were analyzed using the Chi-square test or Fisher’s exact test. Significance was assumed when *P* < 0.05.

## SUPPLEMENTARY MATERIAL

Supplementary Methods and References

Supplementary Figures

Supplementary Tables
