# Supplementary material for: AluYb8 insertion polymorphism in the MUTYH gene impairs mitochondrial DNA maintenance and affects the age of onset of IPF
Source: Aging (Albany NY). 2019 Feb 4;11(3):933–49. doi: 10.18632/aging.101793 (PMC6382421; doi:10.18632/aging.101793)
Supplement: Supplementary Methods and References [file aging-11-101793-s001.pdf]

SUPPLEMENTARY METHODS

Subjects

The enrollment criteria for the IPF patients were based on the guidelines of the American Thoracic Society (ATS)/European Respiratory Society (ERS)/Japanese Respiratory Society (JRS)/Association of Latin American Thorax (ALAT) in 2011 [1]. We collected the following clinical data at our institution: age, gender, past medical history, smoking history, occupational exposure history, physical examination findings, laboratory results, and high-resolution computed tomography (HRCT) scans. Respiratory specialists diagnosed the recruited IPF patients based on clinical features and HRCT evaluation. Patients with usual interstitial pneumonia of known causes, including collagen vascular disease and environmentally induced pneumonia, were excluded from this study. We also collected pulmonary function test data for the hospitalized IPF patients. The physiological assessment included measurements of the forced vital capacity (FVC), forced expiratory volume in the first second (FEV1) and single breath diffusing capacity for carbon monoxide according to the ATS recommendations. We conducted a continuous follow-up of the IPF patients. Overall survival was defined as the time from the first diagnosis until death from any cause. However, 67 of the 277 patients were lost to follow up (non-response).

All recruited healthy controls were free from other clinical diseases, including acute inflammation, cancer, cardiovascular diseases, diabetes and any neurological or psychiatric disorders, as assessed by their medical history, physical examination and blood chemistry.

Quantification of the mtDNA content by real-time quantitative PCR

We used real-time quantitative PCR (RT-qPCR) to determine the mtDNA content relative to that of the nDNA by measuring the ratio of the encoding mitochondrial genes *MT-TL1* and *MT-ND1* and the nuclear gene β-actin. The primers used for the mtDNA content assay are listed in the Supplementary Data (Supplementary Table 3). The SYBR kit (Vazyme) and ABI StepOne Real-Time System (Applied Biosystems) were used to measure the mtDNA content. The RT-qPCR was carried out in a 20 μl total reaction volume containing 2 μl (50 ng) of DNA, 10 μl of AceQ® qPCR SYBR® Green Master Mix, 0.4 μl of each primer and 7.2 μl of sterile purified water. All mixtures were subjected to 5 min of pre-denaturation at 95°C, followed by 40 cycles of 10 seconds at 95°C and 30 seconds at 60°C. All samples were analyzed in triplicate. The average threshold cycle number (Ct) values of the nDNA and mtDNA were obtained from each case to determine the relative mtDNA quantities in each blood sample. We used the delta Ct (ΔCt=CtmtDNA-CtnDNA) in the same well as an exponent of 2 ($2^{\Delta Ct}$) to calculate the mtDNA content.

Analysis of the mtDNA copy number by droplet-based digital PCR (ddPCR)

To determine the mtDNA copy number, ddPCR was performed in the present study using samples obtained from the IPF patients and heathy controls. The ddPCR reaction mixtures were prepared in a 20 μl volume and contained 0.1 ng of genomic DNA, 1 × ddPCR supermix, 0.5 μM of each primer (Supplementary Table 3), and 0.25 μM of each probe (5'- FAM CCA TCA CCC TCT ACA TCA CCG CCC TAMRA-3' for *ND-1*) in water-in-oil emulsions using the droplet generator (Bio-Rad). Then, the generated droplets were transferred to a 96-well plate and amplified by a PCR thermocycler instrument. The amplification conditions were 1 cycle for 10 min at 95°C, followed by 40 cycles of 30 s at 94°C and 60 s at 60°C, a final step for 10 min at 98°C and cooling to 10°C. Subsequently, the PCR products were detected by the QX200 droplet reader (Bio-Rad), and data analysis was performed using the Quanta Soft analysis software. One nanogram of human genomic DNA is roughly equal to 300 copies of the haploid genome.

Evaluation of the mtDNA integrity

The primers used for the assay are listed in Supplementary Table 3. Using the PicoGreen fluorescent dye (Invitrogen), the long-range PCR product and the relative amounts of mtDNA/nDNA could be quantified as described previously [2] Each PCR analysis was performed in duplicate.

Analysis of mitochondrial genes and mitochondrial-related gene expression

Peripheral blood mononuclear cells were isolated from whole blood samples from the IPF patients and healthy controls with lymphocyte separation medium. Total RNA was extracted from the isolated PBMCs and harvested cells with RNAiso Plus (Takara) and reverse transcribed to cDNA using the HiScript® II One Step RT-PCR Kit (Vazyme) according to the manufacturer's instructions. The AceQ® qPCR SYBR® Green Kit and ABI Viia 7 Real-Time System (Applied Biosystems) were used to amplify and assess the mRNA expression levels of target genes. The mRNA quantification results were normalized to the reference gene expression level calculated with the $2^{\Delta Ct}$ method.

ATP6 and *COX2* mRNA expression was used to assess the mtDNA transcriptional activity. *POLG, MFN2* and *ATG7* expression was used to evaluate mtDNA replication, mitochondrial fusion and mitophagy, respectively. *GFP* and *neomycin* gene expression was used to detect the reporter gene transcription and transfection efficiencies in the transfected cells, respectively. The primer sequences for these assays are listed in Supplementary Table 3.

Cell culture

The human lung carcinoma epithelial A549 and Chinese hamster ovary (CHO) cell lines were cultured in F12K medium supplemented with 10% (v/v) fetal bovine serum (FBS). The human embryonic lung fibroblast (HELF), human embryonic kidney 293T (HEK293T), human hepatocyte LO2, human prostate cancer DU145 and mouse myoblast (C2C12) cell lines were grown in Dulbecco's Modified Eagle's Medium (DMEM) containing 10% (v/v) FBS. HCT15 colon adenocarcinoma cells were propagated in RPMI-1640 medium with 10% (v/v) FBS. All cultures were supplemented with antibiotics (100 U/ml of penicillin and 100 μg/ml of streptomycin), and the cells were seeded in cell culture flasks and maintained in a humidified incubator at 37°C with 5% (v/v) CO_2.

SUPPLEMENTARY REFERENCES

1. Raghu G, Collard HR, Egan JJ, et al. An official ATS/ERS/JRS/ALAT statement: idiopathic pulmonary fibrosis: evidence-based guidelines for diagnosis and management. Am J Respir Crit Care Med. 2011; 183:788–824. https://doi.org/10.1164/rccm.2009-040GL

2. Guo W, Zheng B, Guo D, et al. Association of AluYb8 insertion/deletion polymorphism in the MUTYH gene with mtDNA maintain in the type 2 diabetes mellitus patients. Mol Cell Endocrinol. 2015;409:33–40. https://doi.org/10.1016/j.mce.2015.03.019
