# Supplementary material for: AluYb8 insertion polymorphism in the MUTYH gene impairs mitochondrial DNA maintenance and affects the age of onset of IPF
Source: Aging (Albany NY). 2019 Feb 4;11(3):933–49. doi: 10.18632/aging.101793 (PMC6382421; doi:10.18632/aging.101793)
Supplement: Supplementary Figures [file aging-11-101793-s002.pdf]

SUPPLEMENTARY FIGURES

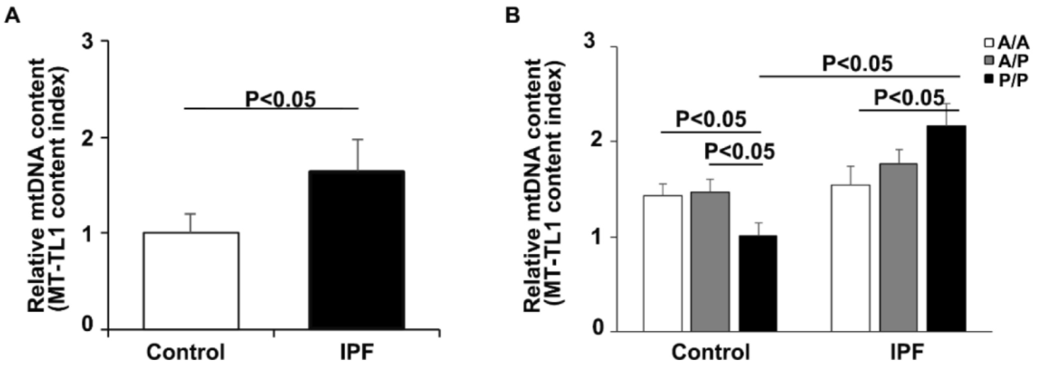

Supplementary Figure 1. Relationship between the *AluYb8MUTYH* genotype and mtDNA content in the IPF patients and healthy controls. (**A**) The *MT-TL1* content index was increased in IPF patients, *P<0.05*. (**B**) In patients with *P/P,* the *MT-TL1* content index was significantly higher than that in healthy controls with *P/P, P<0.05*.

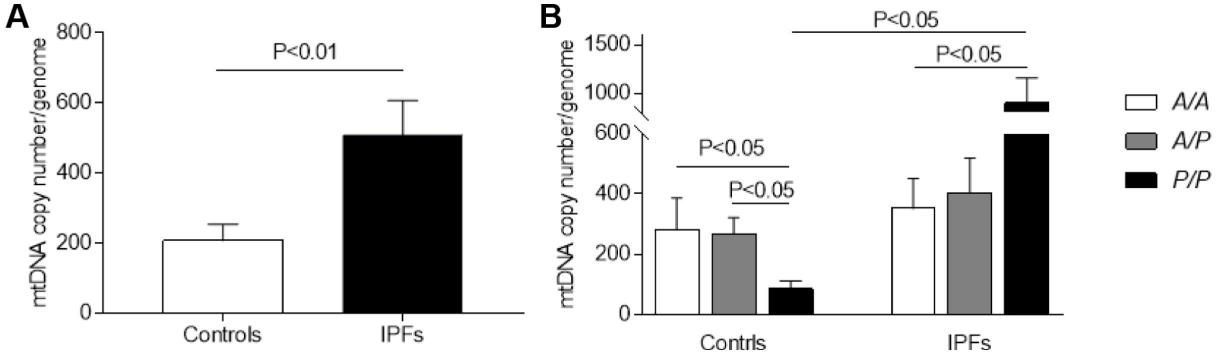

Supplementary Figure 2. Result of the relationship between *AluYb8MUTYH* polymorphism and mtDNA copy number in the healthy control and IPF patient subjects by droplet digital PCR assay. (**A**) The cells of IPF patients have a significantly higher level of mtDNA than that of age-matched controls, *P*<0.01. (**B**) The controls with P/P genotype had decreased levels of mtDNA content when compared to wild-type (A/A) and heterozygote (A/P) controls, and the IPF patients with P/P genotype are just the opposite, *P*<0.05. The result from droplet digital PCR assay is in accordance with the result of relative quantifying mtDNA contents by SYBR green determination. One nanogram of human genomic DNA is roughly equal to 300 copies of the haploid genome.

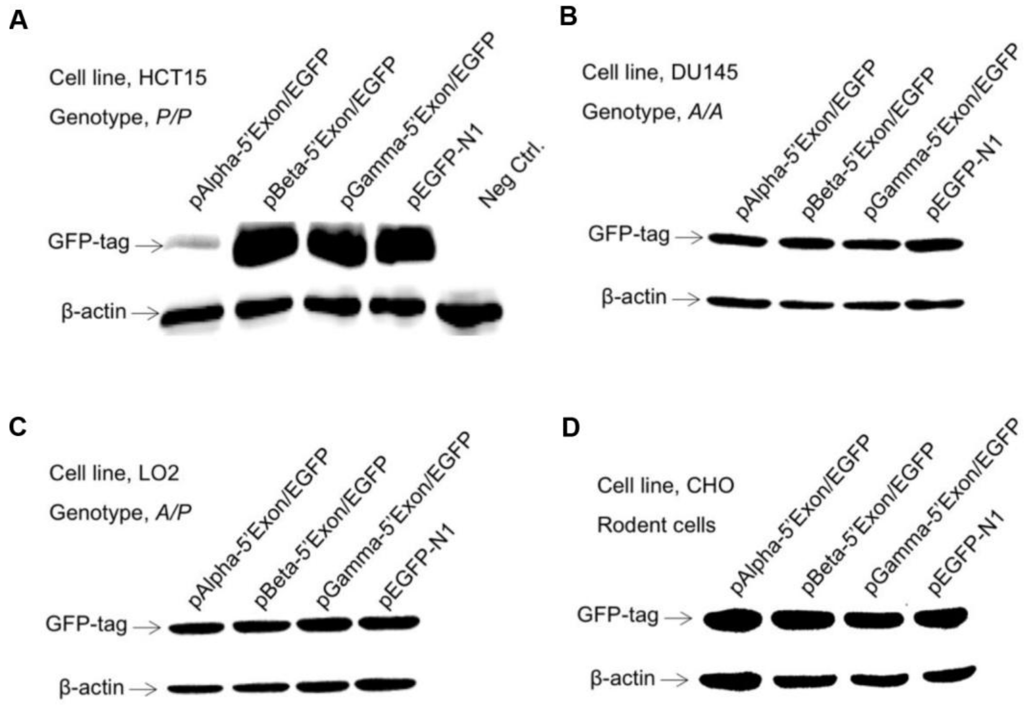

Supplementary Figure 3. Immunoblotting results of GFP expression in reporter gene system. Highly expressed GFP reporter protein of the recombinant pAlpha-5' Exon/EGFP were observed in DU145 (A/A genotype), LO2 (A/P genotype) human cell lines and CHO (Chinese hamster cell line). However, the recombinant pAlpha-5' Exon/EGFP vector expressed GFP reporter protein at a lower level in the human cells (HCT15) with the mutant (P/P) genotype. Additionally, the GFP reporter of the recombinant vectors, pBeta-5'Exon/EGFP and pGamma-5'Exon/EGFP, were highly expressed in all cultured cells. β-*actin* was kept as protein loading control.

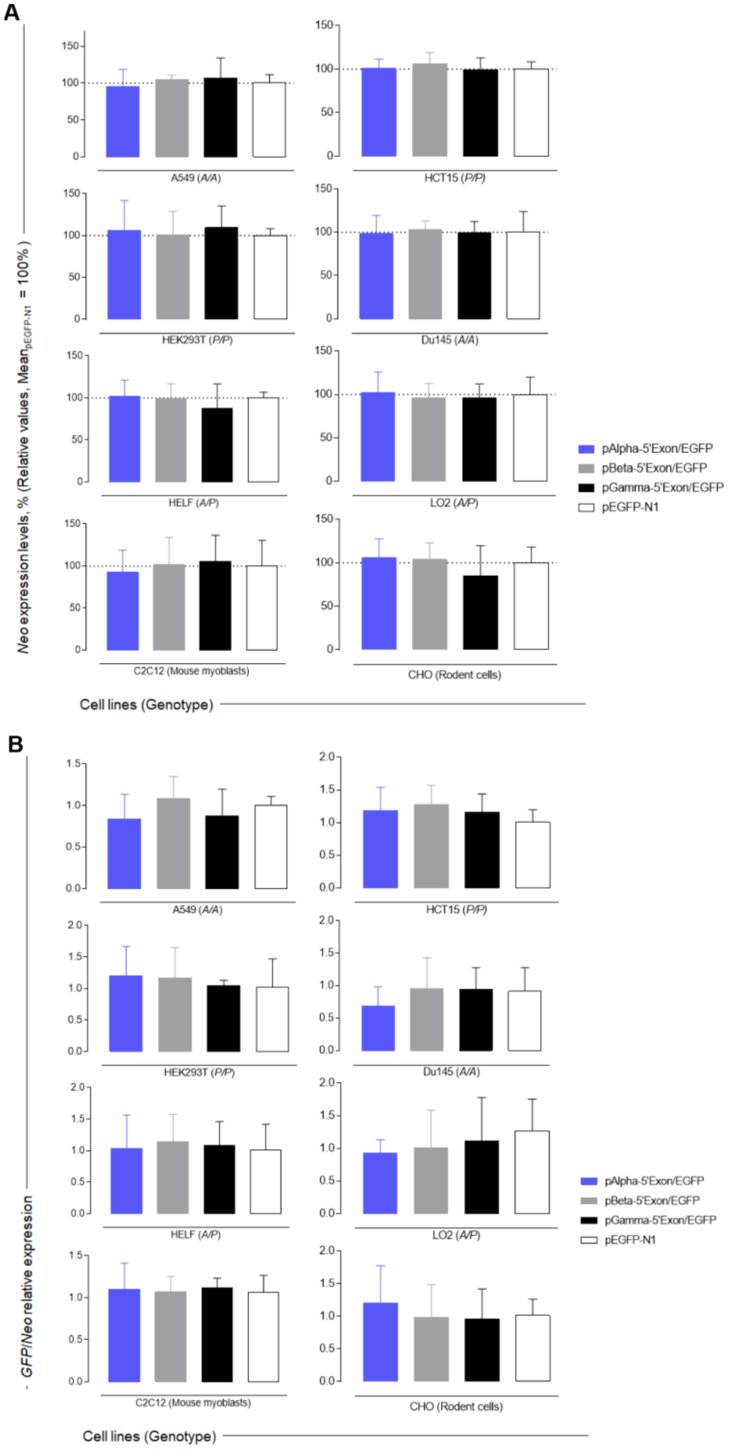

Supplementary Figure 4. The mRNA expression analysis for *GFP* and *Neo* genes of the recombinant vectors in all cultured cells. (**A**) No statistically significant difference in the mRNA expression levels of the embedded *Neo* gene among the cultured cells with different recombinant vectors. The relative expressions of *neomycin* and a host housekeeping gene (β-*actin*) reflect plasmid transfection efficiency of the recombinant vectors in the experimental cell lines. The results indicate there is no significant difference in the transfection efficiency for the different recombinant vectors in all the experimental cell lines under the standard transfection condition. The average value of the *Neo* relative expression (*Neo/*β-*actin*) of cultured cells with the *pEGFP-N1* vector was set to 100. Data are mean ± s.d. (**B**) Among the experimental cell lines with the different recombinant vectors, the difference was not statistically significant in the mRNA expression levels of *GFP* reporter gene (P > 0.05). An embedded *neomycin* encoded gene within the pEGFP-N1 vector was used as the reference for the GFP reporter gene. Data are mean ± s.d.
