# Supplementary material for: AluYb8 insertion polymorphism in the MUTYH gene impairs mitochondrial DNA maintenance and affects the age of onset of IPF
Source: Aging (Albany NY). 2019 Feb 4;11(3):933–49. doi: 10.18632/aging.101793 (PMC6382421; doi:10.18632/aging.101793)
Supplement: Supplementary Tables [file aging-11-101793-s003.pdf]

SUPPLEMENTARY TABLES

Supplementary Table 1. The demographic characteristics of the study subjects.

	Healthy controls	IPF patients
Number	810	277
Age*	53.96±14.08	61.38±12.60
Male	421	240

*** The data represents the mean ± SD.**

Supplementary Table 2. Allele frequency of *AluYb8MUTYH* in IPF and healthy control.

	Allele frequency		*P* value	OR(95%CI)
	IPF	Healthy control		
A	325 (58.7%)	892 (55.1%)	0.15	0.86
P	229 (41.3%)	728 (44.9%)		(0.71-1.05)

Supplementary Table 3. Primers used in the present study.

	Primers
β-actin-F	GATCAAGATCATTGCTCCTCCTG
β-actin-R	CCTCGGCCACATTGTGAACT
MT-TL1-F	CACCCAAGAACAGGGTTTGT
MT-TL1-R	TGGCCATGGGTATGTTGTTA
MT-ND1-F	AACATACCCATGGCCAACCT
MT-ND1-R	AGCGAAGGGTTGTAGTAGCCC
MtDNA-F	TCTAAGCCTCCTTATTCGAGCCGA
MtDNA-R	TTTCATCATGCGGAGATGTTGGATGG
nDNA (β-actin)-F	CATTTACTCTGGGTGATGTCTTTCC
nDNA (β-actin)-R	GGCTATGTCTTAGGGTTGAGGTCTT
ATP6-F	CCTAGCCCACTTCTTACCACA
ATP6-R	GCTTGGATTAAGGCGACAG
COX2-F	CCCCACATTAGGCTTAAAAACAGAT
COX2-R	TATACCCCCGGTCGTGTAGC
POLG-F	TGACCGAGTAGGCAGTGAGT
POLG-R	AGGCCCTTGGTGGCAGCGTA
MFN2-F	GAAGGCTTTCAAGTGAGGAT
MFN2-R	GGTCTTGCCGCTCTTCACG
ATG7-F	TTGAAGTTGTTTGCTTCCGTGAC
ATG7-R	TAGGCAATCTTCAAACTCATAG
UTR1-B-F	AGGAGATCTCAGCCGGAGCCGCGGTGTACAA
UTR1-B-R	CCA CTGCAG CCACAGACGACTCAGGCGGGA
UTR2-B-F	GTTAGATCTTCCCGGGTCCGGCGCGGGGAAGGCA
UTR2_P_R	AAACTGCAGCTCCCGCGAGCTCTAGCGCGCCCGG
UTR3_B_F	CTCAGATCTTCTCGCGGCGGGAACGCGGGGCCT
UTR3_P_R	ACCCTGCAGCCGCGGCCCACGCTGATGAAGAC
GFP-F	AGTGCTTCAGCCGCTACCC
GFP-R	AGTTCACCTTGATGCCGTTC
Neo-F	TGACTGGGCACAACAGACAA
Neo-R	GGCAGGAGCAAGGTGAGATG
β-actin (M)-F	CCAACCGCGAGAAGATGA
β-actin (M)-R	CCAGAGGCGTACAGGGATAG
